# Translation, cross-cultural adaptation and validation of the polish version of the Oxford Shoulder Score in patients undergoing arthroscopic rotator cuff repair

**DOI:** 10.1186/s12955-019-1263-1

**Published:** 2019-12-30

**Authors:** Agnieszka Bejer, Magdalena Szczepanik, Jędrzej Płocki, Daniel Szymczyk, Marek Kulczyk, Teresa Pop

**Affiliations:** 1 Medical College of Rzeszow University, Institute of Health Sciences, ul. Warzywna 1A, 35-310 Rzeszów, Poland; 2 The Holy Family Specialist Hospital, Rudna Mała, Poland; 3 Department of Physiotherapy, University of Information Technology and Management, Faculty of Medicine, Rzeszow, Poland

**Keywords:** Shoulder, Rotator cuff, Oxford shoulder score, Patient-reported outcomes, Reliability, Validity

## Abstract

**Background:**

The Oxford Shoulder Score (OSS) is a simple and reliable, joint-specific, self-reported outcome measure. It can be applied in patients with shoulder disease other than instability. The purpose of this study was to perform a translation, cultural adaptation of the Polish version of the OSS and to evaluate its selected psychometric properties in patients after arthroscopic rotator cuff repair.

**Methods:**

Sixty-nine subjects participated in the study, with a mean age 55.5 (ranging from 40 to 65 years). The OSS has been translated using the widely accepted guidelines. All patients completed the Polish version of OSS (OSS-PL), the short version of the Disabilities of Arm, Shoulder and Hand Questionnaire (QuickDASH), the Short Form-36 v. 2.0 (SF-36) and the 7-point Global Rating of Change Scale (GRC).

**Results:**

High internal consistency of 0.96 was found using Cronbach’s alpha coefficient. Reliability of the OSS resulted in Intraclass Correlation Coefficient (ICC) = 0.99, Standard Error of Measurement (SEM) = 1.14 and Minimal Detectable Change (MDC) = 3.15. The validity analysis showed a moderate (General health r = 0.34) to high (Physical role functioning r = 0.82) correlation between the OSS-PL and SF-36 and a high correlation between the OSS-PL and the QuickDASH (r = − 0.92).

**Conclusions:**

The Polish version of OSS is a reliable and valid, self-reported questionnaire, which can be applied in patients with a rotator cuff tear undergoing reconstruction surgery. The very good psychometric properties of the Polish version of the OSS indicate that it can be used in clinical practice and scientific research.

## Background

Rotator cuff disease is a common disorder which affects 30 to 50% of the population older than 50 years [1]. It includes a wide spectrum of pathological changes ranging from tendinopathy to partial or complete tears [2]. Rotator cuff disease is associated with shoulder pain, loss of function and decreased quality of life. Early surgical repair can be considered for acute tears in all age groups, as well as in case of chronic, reparable tears in young patients (< 65 years old) of substantial size (> 1 cm) without significant, chronic muscle changes [2].

The Oxford Shoulder Score (OSS) was developed by Dawson et al. in 1996. It is a 12-item self-administered questionnaire, primarily designed to evaluate pain and shoulder function for patients undergoing shoulder surgery (excluding shoulder stabilization) [3]. The authors of OSS presented some modification to the scoring system in their article published in 2009 [4]. This clinical measure, as a joint-specific instrument, minimizes the influence of other, concurrent diseases on the outcome score. The OSS is considered to be a quick, simple and reliable patient-reported outcome measure for the English-speaking population. The questionnaire showed good psychometric properties [3].

The OSS is also an internationally used patient-reported outcome measure (PROM). It has been already translated and adapted into different languages, but not into Polish. The OSS questionnaire has been translated into German 2004 [5], Norwegian 2008 [6], Italian 2010 [7], Dutch 2010 [8], Turkish 2011 [9], Korean 2012 [10], Chinese 2015 [11], Persian 2015 [12], Spanish 2015 [13], French 2016 [14], Portuguese 2018 [15].

The aim of this study was to translate and adapt the OSS into Polish, as well as to evaluate its selected psychometric properties (reliability and validity) in patients following arthroscopic rotator cuff repair.

## Methods

### Translation and cross-cultural validity

The OSS has been translated using the guidelines recommended by the Oxford University Innovation to ensure adequate, high standards [16].

**Fig. 1:**
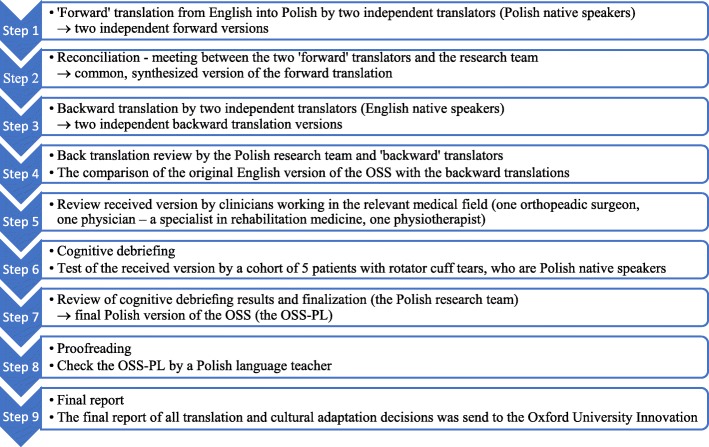
Flowchart of the translation and cultural adaptation process of the OSS from English into Polish

The process of the cross-cultural adaptation of the OSS consisted of nine steps, each of them was documented in a written report (Fig. 1).

Step 1. ‘Forward’ translation from English into Polish by two independent translators (Polish native speakers) → two independent forward versions.

One of the translators had a medical background and knowledge about the concept of the questionnaire and the purpose of the study. The second translator had no medical preparation and was not informed about the concept of the questionnaire and the purpose of the translation.The high standards of the process of accurate translation, corresponding to the original version, allows for some typical Polish language equivalents to be introduced for some original English language terms that would otherwise be difficult to translate.

Step 2. Reconciliation- meeting between the two ‘forward’ translators and the Polish research team →common version of the forward translation.

The team analyzed all items, questions and answers, as well as the instructions for completing the OSS questionnaire. There were some acceptable differences between the two translations, resulting from the many Polish language equivalents which can be used by the translators. The result at this stage was the elaboration of one synthesized version of the ‘forward’ translation.

Step 3. Backward translation by two independent translators (English native speakers) → two independent backward translation versions.

Our back-translators were not familiar with the original version or any other different language versions of the OSS and also they were also not aware of the intended concepts of the questionnaire translation. Only one of the English native speakers was experienced in medical literature.

Step 4. Back translation review.

Comparison of the original English version of the questionnaire with the backward translations was done by the Polish research team and ‘backward’ translators. This team meeting was focused on resolving any remaining problems, discrepancies and ambiguities. There were some corrections introduced, concerning three items - #4, #8 and #12 and one answer in the questionnaire. The main problem resulted from a large number of Polish language equivalents which can be used for ‘extreme’, ‘at the same time’, ‘been troubled’, and matching their best meaning in Polish concerning the concept of the questionnaire item.

Step 5. Review by clinicians working in the relevant medical field.

The assessment of received version was performed by a team of experts composed of one orthopaedic surgeon, one physician – a specialist in rehabilitation medicine – and one physiotherapist with 15 years’ experience working with orthopaedic patients. Their task was to assess the consistency of each question in the original version with the same questioning our version of the questionnaire, i.e. to consider whether both questions assess the same patients’ symptoms or problems. Consistency was assessed on a 6-point scale from 0 to 5, where ‘0’ means that the Polish translation is inadequate, and ‘5’ that it is fully adequate. If the expert scored a question at ‘3’ or less, he was obliged to present an alternative proposal. As a result of work of the expert team, small corrections were made in questions 3, 4 and 10.

Step 6. Cognitive debriefing.

The received version was tested on a cohort of 5 patients (3 women and 2 men) aged from 49 to 57 years, with rotator cuff tears (> 3 months), who are Polish native speakers. They had suitable experience in functioning in everyday life with a painful shoulder, so they were able to reliably assess the accuracy and clarity of the questions and answers in our questionnaire. Their task was to complete the questionnaire and state whether a given item was fully comprehensible or it caused any doubts. All subjects provide answers to all questions of this version of the questionnaire. The answers were then assessed according to a three points scale, where: ‘2’ meant that the question was fully comprehensible, ‘1’ – that the question was only partially comprehensible, and ‘0’ – that the question was totally incomprehensible. In all cases where a question was found to be incomprehensible to a tested subject, he or she was asked to give a reason for the lack of understanding. The patients did not report any difficulties with understanding the questions (mean = 1.98) or the answer options (mean = 2.0) included in our version. Additionally, the graphic layout of our questionnaire was evaluated as very high by the subjects (mean = 2.0).

Step 7. Review of cognitive debriefing results and finalization→ final Polish version of the OSS.

At this stage, no changes of the received version of OSS were introduced by the Polish research team. The final Polish version of the OSS (the OSS-PL) was approved.

Step 8. Proofreading.

In the stage, a Polish language teacher checked the received version of the OSS for any minor errors (spelling, grammatical or other), which could have been omitted during the translation process. There were no such errors reported.

Step 9. Final report.

The final report providing a description of all translation and cultural adaptation decisions and procedures was sent to the Oxford University Innovation.

### Participants

Patients after arthroscopic rotator cuff muscle reconstruction surgery (supraspinatus, infraspinatus, subscapularis and the tendon of biceps brachii – long head tears) were qualified for the study group. All patients who were operated on in 2015 and 2016 in the Department of Orthopedics and Traumatology, The Holy Family Specialist Hospital in Rudna Mala/Rzeszow (Poland), were invited to participate in this study.

The inclusion criteria in the study group were: patients after arthroscopic rotator cuff muscle reconstruction surgery, time from surgery from 3 months to 2 years, adults over 18 years old, Polish native speakers and patient’s written informed consent. The exclusion criteria were as follows: previous surgery of the shoulder girdle and the upper limb, previous injuries of the shoulder complex and the upper limb (luxations, sub-luxations, fractures) and concurrent rheumatic diseases (rheumatoid arthritis, ankylosing spondylitis) and neurological diseases (stroke, multiple sclerosis, Parkinson disease, cervical radiculopathy).

Ethical approval was obtained for the study (reference: No 1/6/2017), and all participants were informed about the purpose and procedures of the study and gave their written consent.

### Measures

#### Oxford Shoulder Score (OSS)

The OSS is a standard, self-reported questionnaire developed for patients with shoulder disease other than instability. It consists of 12 questions concerning pain and disability during the last four preceding weeks, with five categories of possible responses. Responses to each question in the OSS are scored on a scale of 0–4 points, with ‘4’ being the best score. The overall score of the OSS (ranging from 0 to 48) is the sum of the 12 item scores [3, 4, 17].

#### Short Form-36 v. 2.0 (SF-36 v. 2.0)

The SF-36 v. 2.0 is a generic Health Related Quality of Life (HRQOL) questionnaire, which consists of 36 questions, divided into eight sections: vitality, physical functioning, bodily pain, general health, physical role functioning, emotional role functioning, social role functioning, mental health. In each of the questionnaire sections one can obtain a score from 0 to 100. The lower the score means the lower subject’s quality of life. Taking into account the eight dimensions of the SF-36 v. 2.0, two summary scores, one for physical health (PCS – Physical Component Summary) and one for mental health (MCS – Mental Component Summary), can be computed [18, 19].

#### Disabilities of Arm, Shoulder and Hand Questionnaire-Short Version (QuickDASH)

The QuickDASH is a self-report questionnaire for the assessment of functional disability of the upper limbs. It consists of 11 questions concerning: symptoms (3 questions) and influence of the upper limb problems on the subject’s social activities, work and daily living (8 questions). Responses to each question in the QuickDASH are scored on a scale of 1–5 points, with‘1’ being the best score. The overall score of the questionnaire is ranging from 0 to 100, where the higher values indicate a patient’s greater limitation and symptoms intensity. The overall score of the QuickDASH was calculated using original scoring formula: QuickDASH Scoring Formula = ([(sum of *n* responses)/*n*]-1, 25), where *n* represents the number of completed items [20, 21].

### Procedure

Initially, permission to use the OSS in this study was obtained from the license-holder (Oxford University Innovation). The translated version was then applied in a 2-stage survey in order to verify its measurement properties (reliability and validity). During the first assessment all subjects had to complete the following questionnaires: the Polish version of Oxford Shoulder Score (OSS-PL), the Polish short version of Disabilities of Arm, Shoulder and Hand Questionnaire (QuickDASH) and the Polish version of Short Form-36 v. 2.0 (SF-36). The second assessment was conducted after an interval of one to two weeks after the first study. This time the subjects were asked to complete the OSS-PL questionnaire again, as well as to specify if they had experienced any changes in the symptoms and function concerning the operated shoulder, using the 7-point Global Rating of Change Scale (GRC) (1 = much better, 2 = somewhat better, 3 = a little better, 4 = no change, 5 = a little worse, 6 = somewhat worse, 7 = much worse) [22].

The test-retest time interval was assumed according to scientific literature, which indicates that an interval of one to two weeks is adequate and reasonable in such a study [23, 24].

### Statistical analysis

All statistical analyses were conducted using the Statistica 10.0 software. The level of statistical significance was assumed at α < 0.05. Normal distribution of the results was verified using the Shapiro-Wilk test. A non-parametric Wilcoxon test was used for basic statistical analysis. The sample size was based on the general recommendations of Altman, being at least 50 subjects for such a study [25].

### Reliability

#### Internal consistency

Internal consistency is a measure of the correlation between different items on the same test (or the same subscale of the test). It is used to measure whether different items intended to measure the same domain in the test, produce similar scores. It was calculated using Cronbach’s alpha coefficient, based on the data obtained from the first examination (*N* = 69). Internal consistency of the scale is considered to be acceptable when Cronbach’s alpha coefficient value is ≥ 0.7 [23, 24, 26]. We hypothesized that Cronbach’s alpha coefficient value for the OSS-PL will be ≥ 0.7.

#### Reliability

Reliability concern the degree to which repeated measurements in the group of the same persons and same conditions (test-retest) provide similar results, where no change has occurred. To assess the reliability of the OSS-PL, the Intraclass Correlation Coefficient (ICC) with a 95% confidence interval (CI) was used. It was calculated based on the data from 57 subjects who had completed the OSS-PL twice. According to Terwee et al. the reliability of an instrument is acceptable when the ICC is at least 0.70 [24, 26]. We hypothesized that ICC for the OSS-PL will be ≥ 0.7.

### Standard error of measurement and minimal detectable change

Reliability concerns the degree to which patient conditions can be distinguished from each other despite measurement error. It concerns the absolute measurement error, i.e., how close the scores on repeated measures are. A small measurement error is required for evaluation purposes in order to distinguish clinically important changes from measurement error [24]. The estimates of Standard Error of Measurement (SEM) and Minimal Detectable Change (MDC) were calculated, based on the same data from 57 subjects who had completed the OSS-PL twice.

The SEM was calculated using the formula: SEM = SD √(1-R), where SD represents SD of the sample and R the reliability parameter (ICC).

The MDC is the minimum amount of change in a patient’s score that ensures the change is not the result of a measurement error. The MDC was calculated using the formula: MDC=SEMx1.96x √2,

where 1.96 derives from the 0.95% CI of no change, and √2 shows two measurements assessing the change [24, 26, 27].

### Validity

#### Construct validity- hypotheses testing

Construct validity refers to the extent to which a test measures the intended construct, related to other measures, in a manner that is consistent with theoretical hypotheses concerning the concepts that are being measured [24, 26]. In order to evaluate the construct validity of the Polish version of the OSS, the Spearman correlation coefficient (SCC) was calculated between the total and specific domain scores of the OSS-PL, the specific questionnaire for the assessment of the upper limb function (the QuickDASH) and the general quality of life questionnaire (the SF-36). Correlation coefficients used to assess validity were classified as follows: r < 0.30 = low, 0.30 < r < 0.70 = moderate, r > 0.70 = high [28]. We expected that correlations between the OSS and related subscales of the SF-36 and the QuickDASH would be the strongest. We hypothesized that:

- correlation between the OSS-PL and the SF-36: Bodily pain, Physical functioning, Physical role functioning and total of PCS component of the SF-36 would be strong and positive,
- correlation between the OSS-PL and the SF-S6: General health, Vitality, Social role functioning, Emotional role functioning, Mental health and total of MCS component of the SF-36 would be moderate or low and positive,
- correlation between the OSS-PL and the QuickDASH would be strong and negative.

## Results

### Description of population

After screening the database of The Holy Family Specialist Hospital in Rudna Mała (Poland), 111 subjects operated in 2015–2016 for a rotator cuff tear and therefore meeting the inclusion criteria, were invited to participate in this study. The subjects were contacted by telephone and informed about the research objective and procedure. They were asked to complete the questionnaires, which were sent to them by post, in accordance with the enclosed testing procedure instructions. 37.8% (*N* = 42, 24 males, 18 females, mean age = 56.5) of the initially enrolled subjects refused to participate in this study. 62.2% of the subjects (*N* = 69, 49 males, 20 females, mean age = 55.5) completed and sent back the questionnaires. Each of the subjects provided answers for all questions included in the OSS-PL. Patient sex, age, operated shoulder side and time from surgery were also recorded (Table 1).

**Table 1:** Characteristics of the study population (*n* = 69) and population that refused to participate in the study (*n* = 42)

	Participants (n = 69)		Non-participants (n = 42)	
	Number (%)	Mean (range)	Number (%)	Mean (range)
Gender				
Male	49 (71)		24 (57)	
Female	20 (29)		18 (43)	
Age (years)		55.5 (40–65)		56.5 (38–75)
Operated shoulder side:				
Right	58 (84)		33 (79)	
Left	11 (16)		9 (21)	
Time from surgery		1.4 years (0.5–2 years)		1.2 (3 months-2 years)
Diagnosis (single/multiple muscle tear):				
4 muscles	7 (10.1)		5 (12)	
3 muscles	22 (31.9)		6 (14)	
2 muscles	24 (34.8)		13 (31)	
1 muscles	16 (23.2)		18 (43)	

### Evaluation of psychometric properties of OSS-PL

Table 2 shows baseline mean values for the OSS-PL, the SF-36 and the QuickDASH (Table 2).

**Table 2:** The OSS-PL, the SF-36, the QuickDASH results

Questionnaire	$\overline{x}$±SD	Range
OSS-PL	36.0 ± 10.4	15–48
SF-36		
Physical Functioning	77.5 ± 19.5	15.0–100.0
Physical Role Functioning	58.8 ± 26.3	6.3–100.0
Bodily Pain	64.9 ± 25.2	22.5–100.0
General Health	62.0 ± 13.9	25.0–90.0
Vitality	63.0 ± 18.2	18.8–100.0
Social Role Functioning	76.3 ± 22.5	25.0–100.0
Emotional Role Functioning	75.0 ± 24.8	25.0–100.0
Mental Health	71.8 ± 17.9	20.0–100.0
PCS	63.3 ± 14.7	26.0–85.7
MCS	70.6 ± 18.2	21.4–100.0
QuickDASH	28.6 ± 24.7	0.0–84.1

*SD* Standard Deviation, *OSS-PL* Polish version of Oxford Shoulder Score,

*SF-36* Short Form-36, *PCS* Physical Component Summary, *MCS* Mental Component Summary

### Reliability

#### Internal consistency

Internal consistency was excellent with overall Cronbach’s alpha value =0.96. All items showed strong correlations with the total score (ranged from 0.80 to 0.93) (Table 3).

**Table 3:** Internal consistency of OSS-PL

Item	Mean score ± SD	Item-total score correlation		Cronbach’s alpha
		*R*	*P*	
1	2.77 ± 0.91	0.90	0.0000	0.96
2	3.01 ± 0.87	0.90	0.0000	0.93
3	3.41 ± 0.73	0.80	0.0000	0.97
4	3.45 ± 0.78	0.85	0.0000	0.94
5	3.14 ± 0.99	0.84	0.0000	0.94
6	3.17 ± 1.10	0.82	0.0000	0.97
7	2.96 ± 1.16	0.89	0.0000	0.96
8	2.61 ± 1.11	0.88	0.0000	0.96
9	2.67 ± 1.20	0.93	0.0000	0.96
10	3.19 ± 1.05	0.89	0.0000	0.96
11	2.77 ± 0.99	0.86	0.0000	0.96
12	2.90 ± 1.15	0.84	0.0000	0.97

*SD* standard deviation, *r* value of Spearman correlation coefficient, *p* probability value

### Reliability

For the purpose of the test-retest reliability and SEM and MDC assessment the subjects completed the OSS-PL twice. The average time between the test-retest study was 10 days (ranging from 7 to 14 days). 57 out of 69 patients completed the OSS twice to assess its reliability. Four subjects were excluded due to changes in their symptoms and function concerning the operated shoulder reported in the GRC scale in the second study. Eight subjects did not send back the second questionnaire. There was no statistically significant difference observed between the test and re-test assessment (*p* = 0.1080). The value of ICC was very high (0.99, CI ranged from 0.98 to 0.99) (Table 4).

**Table 4:** Test-retest of the OSS-PL

	ICC (95% CI)	SEM	MDC
OSS-PL (*n* = 57)	0.99 (0.98–0.99)	1.14	3.15

*OSS-PL* Polish version of Oxford Shoulder Score, *ICC* Intraclass Correlation Coefficient,*95% CI* 95% Confidence Interval, *SEM* Standard Error of Measurement, *MDC* Minimal Detectable Change

#### Standard Error of Measurement and Minimal Detectable Change

The values of SEM and MDC for OSS-PL are presented in Table 4.

### Validity

#### Construct validity - hypotheses testing

The OSS-PL was significantly correlated with all subscales of the SF-36 and the QuickDASH. The value of correlation between the OSS-PL and the SF-36 ranged from moderate (General health r = 0.34) to high (Physical role functioning r = 0.82) and high between the OSS-PL and the QuickDASH (r = − 0.92) (Table 5).

**Table 5:** Correlation between the OSS-PL and the SF-36, and the QuickDASH

	OSS-PL	
SF-36	*R*	*p*
Physical Functioning	0.81	0.0000
Physical Role Functioning	0.82	0.0000
Bodily Pain	0.81	0.0000
General Health	0.34	0.0044
Vitality	0.54	0.0000
Social Role Functioning	0.66	0.0000
Emotional Role Functioning	0.62	0.0000
Mental Health	0.49	0.0000
PCS	0.82	0.0000
MCS	0.62	0.0000
QuickDASH	−0.92	0.0000

*OSS-PL* Polish version of Oxford Shoulder Score,*SF-36*Short Form-36, *r* value of Spearman correlation coefficient, *p* probability value, *PCS* Physical Component Summary, *MCS* Mental Component Summary

## Discussion

There are numerous patient, self-reported questionnaires dedicated for patients witch shoulder diseases. The majority of them were created in English-speaking countries therefore their application in different countries or communities requires translation, cross-cultural adaptation and validation of these instruments.

Because the evaluation of the psychometric properties of the OSS conducted by Dawson and other researchers confirmed its high internal consistency, reliability, validity and responsiveness, we decided to adapt the OSS into Polish [3, 5–15]. Additionally, the OSS is easy to administer and not time-consuming – completion of the whole questionnaire takes from about one to seven minutes [7]. The questions are clearly defined, therefore patients do not require instructions to answer to all questions. In contrast to similar tools, the OSS was designed not only for evaluation of the main symptoms and limitations during activities of daily living in patients with a wide spectrum of shoulder disorders (excluding instability) but also their quality of life. The possibility of using of this questionnaire by clinicians (medical doctors, physiotherapists) allows for better treatment planning and monitoring in this group of patients in Poland. The purpose of this study was to translate the OSS into Polish, to adapt it and to evaluate its psychometric properties in patients diagnosed with a rotator cuff tear after its reconstruction.

The forward and back translation and adaptation procedure revealed no major content \or linguistic problems. The process of translation and adaptation was performed according to the guidelines of the Oxford University Innovation [16].

All questionnaires of the OSS-PL, which were sent to subjects in our study group, for the purpose of the assessment of its psychometric properties, were complete.

Results of our study confirm all of the hypothesis. The OSS-PL presented high internal consistency (overall Cronbach’s alpha = 0.96, ranged from0.93 to 0.97). It showed very high test-retest reliability (Total ICC = 0.99, 95%CI ranged from 0.98 to 0.99). The results of the OSS-PL validity assessment revealed strong, positive correlation between the OSS-PL and the SF-36 PCS and moderate, positive correlation between the OSS-PL and the SF-36 MCS. Similarly, the correlation between the OSS-PL and the QuickDash were also strong and negative, as it was hypothesised.

The results of the assessment of the psychometric properties of OSS-PL were similar to the original validation study and other studies conducted in different countries [3, 5–15]. It confirmed that the Polish version of the OSS is reliable, repeatable and a valid tool, which can be used in the evaluation of patients with rotator cuff tears. Internal consistency was excellent (Cronbach’s alpha 0.97), and similar or higher than in other validation studies [3, 5–15]. According to the quality criteria of Terwee et al., the interval chosen between test and retest was long enough to ensure that the patient forgets the questions and answers, but that their health condition did not change significantly [24]. An interval of about two weeks between test and retest assessment is also recommended by Consensus-based Standards for the selection of health Measurement Instruments (COSMIN) [26].To assess reliability, similarly to French, Portugal, Persian (Iran), Dutch, Chinese, Spanish and Korean validation studies, the ICC was used. Our result was very high (0.99) and comparable with them (ICC 0.91,0.92, 0.93, 0.97, 0.97, 0.98, 0.9 respectively) [8, 10–15]. Only in the Norwegian study the obtained ICC value was lower (0.83) [6]. In other studies the Pearson correlation coefficient was applied to assess reliability, with results showing high reliability of the OSS [7, 9]. As expected, the strongest correlations were observed between the OSS-PL and domains of the SF-36 concerning Physical Functioning, Bodily Pain and Physical Role Functioning as well as between the OSS-PL and the QuickDASH. These results indicated and confirmed proper construct validity. SF-36 was used also in original validation studies in Germany, Italy, Turkey and Korea. Although the value of correlation obtained in those studies was lower than in ours [5, 7, 9, 10]. Ekeberg et al. have conducted a study concerning agreement, reliability and validity of the OSS, the WORC and the SPADI in group of patients with rotator cuff disease. Their research showed a high level of agreement and reliability and small differences between the results of all questionnaires used in their study [6].

Booker et al. reviewed the scientific articles regarding self-reported questionnaires used to assess patients with shoulder disorders. They noticed that in Europe the Constant Score (CS), the DASH and the OSS are the most commonly used, while in the USA, the American shoulder and elbow surgeons standard shoulder assessment form (ASES), the Simple shoulder test (SST) and the UCLA are the most popular [29]. Christiansen et al. compared responsiveness and minimal clinically important change (MCIC) of the modified the CS and the OSS. A group of 126 patients after arthroscopic decompression surgery for subacromial impingement syndrome, reporting problems with returning to their usual daily activities, participated in their study. The evaluation of the patients was conducted at baseline and after 3 months and the OSS, the CS, and the European Quality of Life-5 Dimensions-3 Level (EQ-5D-3 L) index were used. This study showed that the CS and the OSS were both suitable for assessing and detecting of changes in the level of a patient’s daily activities after decompression surgery [30]. One of the obvious advantage of the condition-specific PROMs such as the OSS, compared to standard clinical assessments, such as the Constant Score, is that PROM scan be self-completed anywhere and don’t require hospital visits and the are not clinicians’ time consuming.

### Study strengths and limitations

The major limitation of our study concerns the study group, which consisted only of patients with the rotator cuff tear undergoing reconstruction surgery. Because the OSS was originally developed as a self-reported questionnaire to evaluate patients with a wide spectrum of shoulder disease, excluding instability, we would like to highlight the need to check its psychometric properties in patients with other shoulder conditions, like impingement syndrome, biceps tendonitis, or frozen shoulder. Moreover the current study did not include the assessment of the responsiveness of the OSS-PL, which suggest the need for future studies including this aspect of the OSS-PL.

The strengths include the use of standardized methods for both translation’s process and the evaluation of the psychometric properties of the OSS-PL. Further strength is the agreement between the results of our study and the results concerning English version of the OSS, as well as those reported in the other authors OSS linguistic adaptation and validation studies.

## Conclusion

This study indicated that the Polish version of the OSS is a reliable and valid, self-reported questionnaire, which can be applied to patients with rotator cuff tears undergoing reconstruction surgery. The very good psychometric properties of the OSS-PL allow for its use in clinical practice, as well as in national and international research projects, concerning patients after rotator cuff reconstruction surgery.

## Data Availability

The datasets used and/or analyzed during the current study are available from the corresponding author on reasonable request.

## Abbreviations

ASES: American Shoulder and Elbow Surgeons Standard Shoulder Assessment Form
CI: Confidence Interval
CS: Constant Score
DASH: Shoulder Rating Scale, the Disabilities of the Arm, Shoulder and Hand Index
GRC: Global Rating of Change Scale
HRQOL: Health Related Quality of Life
ICC: Intraclass Correlation Coefficient
MCIC: Minimal Clinically Important Change
MCS: Mental Component Summary
MDC: Minimal Detectable Change
OSS: Oxford Shoulder Score
OSS-PL: Oxford Shoulder Score – Polish version
p: Probability Value
PCS: Physical Component Summary
PROM: Patient-Reported Outcome Measure
QuickDASH: Disabilities of Arm, Shoulder and Hand Questionnaire-short version
r: Value of Spearman Correlation Coefficient
SCC: Spearman Correlation Coefficient
SD: Standard Deviation
SEM: Standard Error of Measurement
SF-36 v. 2.0: Short Form-36 v. 2.0
SPADI: Shoulder Pain and Disability Index
SST: Simple Shoulder Test
UCLA: University of California Los Angeles
USA: United States of America
WORC: Western Ontario Rotator Cuff
